# 102 Vaping of Vitamin E Acetate Causes Acute Lung Injury in a Dose-dependent Manner in Sheep

**DOI:** 10.1093/jbcr/irac012.105

**Published:** 2022-03-23

**Authors:** Kento Homma, Kazuki Hashimoto, Nikolay Bazhanov, John Salsbury, Maret G Traber, Perenlei Enkhbaatar

**Affiliations:** Department of Anesthesiology, University of Texas Medical Branch, Galveston, Texas; Department of Anesthesiology, University of Texas Medical Branch, Galveston, Texas; Department of Anesthesiology, University of Texas Medical Branch, Galveston, Texas; Department of Anesthesiology, University of Texas Medical Branch, Galveston, Texas; Linus Pauling Institute Oregon State University, Corvallis, Oregon; Professor, Department of Anesthesiology, Director, Translational Intensive Care Unit Charles Robert Allen Professor in Anesthesiology The University of Texas Medical Branch, Galveston, Texas

## Abstract

**Introduction:**

The Centers for Disease Control and Prevention and the Food and Drug Administration have reported an increasing number of clinical cases of pulmonary injury following the use of e-cigarette/vaping products. Although the causative factors for the national outbreak of electronic-cigarette, or vaping product use-associated lung injury (EVALI) has not been established, CDC reported that vitamin E acetate (VEA) is strongly linked to the EVALI outbreak. In this study, we tested the hypothesis that VEA vaping causes acute lung injury in a dose-dependent manner in a sheep model.

**Methods:**

Sheep were surgically prepared under anesthesia and analgesia with multiple vascular catheters (pulmonary arterial, left atrial, and femoral arterial). To assess pulmonary edema, the mediastinal lymph node vessel draining the lung was cannulated. After a 5-day surgical recovery, a tracheostomy tube and urinary catheter were placed. Then, the sheep were placed on a mechanical ventilator and VEA vaping was immediately started in the following groups: (1) vaped with glycerol (n=1); (2) vaped with 0.4mg of VEA (n=2); (3) vaped with 0.6mg of VEA (n=1); (4) vaped with 0.8mg of VEA (n=7); and (5) not injured, not treated (Sham, n=6). Sheep were resuscitated with lactated Ringer’s solution (4mL/kg/hr). Sheep in a conscious state was monitored with 4 hrs intervals for cardiopulmonary variables for 48 hrs.

**Results:**

Pulmonary gas exchange, represented by the PaO2/FiO2 ratio, was unchanged in sham, glycerol, and 0.4 mg VEA groups. In the 0.6 VEA group, the PaO2/FiO2 ratio was decreased from 42 to 48 hrs, while in the 0.8 mg VEA group, it was strongly decreased starting at 24 hrs and remained low throughout the remaining period. Lung lymph flow, an index of pulmonary microvascular permeability, was increased more than 2-fold in 0.6 and 0.8 mg VEA groups. Lung compliance also tended to decrease in all VEA groups.

**Conclusions:**

VEA vaping causes acute lung injury in a dose-dependent manner in sheep. Further studies should investigate underlying mechanistic aspects that cause increased microvascular permeability.

